# Supplementary material for: Unique features of KGN granulosa-like tumour cells in the regulation of steroidogenic and antioxidant genes
Source: PLoS One. 2024 Aug 7;19(8):e0308168. doi: 10.1371/journal.pone.0308168 (PMC11305538; doi:10.1371/journal.pone.0308168)
Supplement: S1 Table — (PDF) [file pone.0308168.s001.pdf]

Unique features of KGN granulosa-like tumour cells in the regulation of steroidogenic and antioxidant genes

Feng Tang, Katja Hummitzsch and Raymond J Rodgers*

School of Biomedicine, Robinson Research Institute, The University of Adelaide, Adelaide, SA 5005, Australia

S1Table. Primers for quantitative RT-PCR

Gene	Gene Name	Forward Primer (5'-3')	Reverse Primer (5'-3')	Amplicon size [bp]
GAPDH	glyceraldehyde-3-phosphate dehydrogenase	TCCTCTGACTTCAACAGCGAC	GTGGTCCAGGGGTCTTACTC	176
POR	cytochrome p450 oxidoreductase	TTTCGCTCATCGTGGGTCTC	GTCTGGGAGCCGTAGAACAC	172
CYP19A1	cytochrome P450 family 19 subfamily A member 1	TCGAGTTTTTCCCCAAACCCA	GGCGATGTACTTTCCTGCAC	157
FDX1	ferredoxin 1	AACCCTGGCTTGTTCAACCT	TGGCATCAGCCACTGTTTCA	200
FDXR	ferredoxin reductase	CCTGGAGAGAACGGACATCAC	AGCCACACTGTCTTCACTCG	72
CYP11A1	cytochrome P450 family 11 subfamily A member 1	GGGAGACAGCAAGATGTCCTT	GCGTGCCATCTCATACAAGT	115
GPX1	glutathione peroxidase 1	CAACCAGTTTGGGCATCAG	TCTCGAAGAGCATGAAGTTGG	126
GPX2	glutathione peroxidase 2	ATTGAGAATGTGGCTTCGCTC	ACCCCCAGGACGGACATAC	183
GPX3	glutathione peroxidase 3	AAGTCGAAGATGGACTGCCA	TGGTGCAAGCTCTTCCTGTAG	195
GPX4	glutathione peroxidase 4	ATTGGTCGGCTGGACGAG	TCGATGTCCTTGGCGGAAAA	199

GPX5	glutathione peroxidase 5	CACCTACTGTGGTCTGACAGC	GGTTGCAGGGAAAGCCCAAC	98
GPX6	glutathione peroxidase 6	CTCAACGGCGAGGAGTACAT	GGCCAACACAATGACACCAA	153
GPX7	glutathione peroxidase 7	TCAACATCCGGGGCAAACTG	GGGAAGGCGAGCACGTTAAA	163
GPX8	glutathione peroxidase 8	TCAGCGTGTTGGCTTTTCCC	TTCCACCTTGGTTCCTTCTTTG	193
LRP8	LDL receptor related protein 8	CGCACTCTCTTCAGCCGTAA	ACACCGTTGAGCCCAGATTT	125
SOD1	superoxide dismutase 1	AGAGGCATGTTGGAGACTTGG	TCCAGCGTTTCCTGTCTTTGT	191
SOD2	superoxide dismutase 2	AGAAGTACCAGGAGGCGTTG	AGTCACGTTTGATGGCTTCCA	174
CAT	catalase	CTTCGACCCAAGCAACATGC	GCGGTGAGTGTCAGGATAGG	91
GSR	glutathione-disulfide reductase	GGAGGTGCTGAAGTTCTCCCA	CGGTTTGAATCCCCAGTTTGT	194
TXN	thioredoxin	TTCCATCGGTCCTTACAGCC	ACGTTGGAATACTTTTCAGAGAG G	199
TXN2	thioredoxin 2	TGAGACACCAGTGGTTGTGG	ACTCAATGGCGAGGTCTGTG	152
TXNRD1	thioredoxin reductase 1	AGAGCGAAAGGTGGTCGAGT	AGTCTGCCCTCCTGATAAGC	191
TXNRD2	thioredoxin reductase 2	AAGATGCCCCCAACTATGGC	CCGTGTGCTCGTCAACAAAG	177
TXNRD3	thioredoxin reductase 3	TTGTTGTCCCGTCACCTCAG	AGTCACATAATGCCTGCCCC	120
PRDX1	peroxiredoxin 1	CCCCACGGAGATCATTGCTT	CGCTTCGGGTCTGATACCAA	174
PRDX2	peroxiredoxin 2	AAGGGTGTCCTTCGCCAGAT	ACGTTGGGCTTAATCGTGTC	158
PRDX3	peroxiredoxin 3	ATTTCCCGAGACTACGGTGTG	GAGGGTTTCTTCCACGCTTC	138

PRDX4	peroxiredoxin 4	GCTTTTGGCGACAGACTTGA	TCCTCCTTGTCTTCGAGGGG	117
PRDX5	peroxiredoxin 5	ACGCTCAGCGGGCTATATACT	GGCTCCCCTTCAAACACCTC	186
PRDX6	peroxiredoxin 6	ACCACTGGCAGGAACTTTGAT	AGGACCATCACACTATCCCCA	116
