# Supplementary material for: Unique features of KGN granulosa-like tumour cells in the regulation of steroidogenic and antioxidant genes
Source: PLoS One. 2024 Aug 7;19(8):e0308168. doi: 10.1371/journal.pone.0308168 (PMC11305538; doi:10.1371/journal.pone.0308168)
Supplement: S3 Table — (PDF) [file pone.0308168.s003.pdf]

Unique features of KGN granulosa-like tumour cells in the regulation of steroidogenic and antioxidant genes

Feng Tang, Katja Hummitzsch and Raymond J Rodgers*

School of Biomedicine, Robinson Research Institute, The University of Adelaide, Adelaide, SA 5005, Australia

S3 Table. The normalised expression values (mean ± SEM) of extremely low-expression glutathione peroxidases (*GPX2, GPX4-7*).

Gene	DMSO control	1mM dbcAMP	Ethanol control	10µM forskolin
GPX2	NA	NA	NA	NA
GPX4	7.96E-05 ± 1.12E-05	4.56E-05 ± 3.49E-06	3.77E-05 ± 5.44E-06	4.29E-05 ± 4.50E-06
GPX5	2.42E-05 ± 4.40E-06	1.63E-05 ± 3.40E-06	3.35E-05 ± 9.09E-06	1.63E-05 ± 3.72E-06
GPX6	NA	NA	NA	NA
GPX7	1.36E-05 ± 7.36E-06	6.66E-06 ± 2.06E-06	2.31E-05 ± 4.90E-06	1.35E-05 ± 1.50E-06

* NA means the mRNA expression of the gene was too low to be detected within 40 PCR cycles; thereby no normalised expression values could be calculated (n = 5).
